# Modeling the transboundary risk of feed ingredients contaminated with porcine epidemic diarrhea virus

**DOI:** 10.1186/s12917-016-0674-z

**Published:** 2016-03-12

**Authors:** Scott Dee, Casey Neill, Aaron Singrey, Travis Clement, Roger Cochrane, Cassandra Jones, Gilbert Patterson, Gordon Spronk, Jane Christopher-Hennings, Eric Nelson

**Affiliations:** Pipestone Applied Research, Pipestone Veterinary Services, 1300 Box, 188 Hwy 75 S, Pipestone, MN 56164 USA; Animal Disease Research and Diagnostic Laboratory, South Dakota State University, Brookings, SD USA; Department of Grain Science, Kansas State University, Manhattan, KS USA; Center for Animal Health and Food Safety, University of Minnesota, St. Paul, MN USA

**Keywords:** Transboundary, Porcine, Epidemic, Diarrhea, Virus, Antimicrobial, Ingredient, Lysine, Soybean meal, Choline

## Abstract

**Background:**

This study describes a model developed to evaluate the transboundary risk of PEDV-contaminated swine feed ingredients and the effect of two mitigation strategies during a simulated transport event from China to the US.

**Results:**

Ingredients imported to the USA from China, including organic & conventional soybeans and meal, lysine hydrochloride, D-L methionine, tryptophan, Vitamins A, D & E, choline, carriers (rice hulls, corn cobs) and feed grade tetracycline, were inoculated with PEDV. Control ingredients, and treatments (ingredients plus a liquid antimicrobial (SalCURB, Kemin Industries (LA) or a 2 % custom medium chain fatty acid blend (MCFA)) were tested. The model ran for 37 days, simulating transport of cargo from Beijing, China to Des Moines, IA, US from December 23, 2012 to January 28, 2013. To mimic conditions on land and sea, historical temperature and percent relative humidity (% RH) data were programmed into an environmental chamber which stored all containers. To evaluate PEDV viability over time, ingredients were organized into 1 of 4 batches of samples, each batch representing a specific segment of transport. Batch 1 (segment 1) simulated transport of contaminated ingredients from manufacturing plants in Beijing (day 1 post-contamination (PC)). Batch 2 (segments 1 and 2) simulated manufacturing and delivery to Shanghai, including time in Anquing terminal awaiting shipment (days 1–8 PC). Batch 3 (segments 1, 2 and 3) represented time in China, the crossing of the Pacific and entry to the US at the San Francisco, CA terminal (day 1–27 PC). Batch 4 (segments 1–4) represented the previous events, including transport to Des Moines, IA (days 1–37 PC). Across control (non-treated) ingredients, viable PEDV was detected in soybean meal (organic and conventional), Vitamin D, lysine hydrochloride and choline chloride. In contrast, viable PEDV was not detected in any samples treated with LA or MCFA.

**Conclusions:**

These results demonstrate the ability of PEDV to survive in a subset of feed ingredients using a model simulating shipment from China to the US. This is proof of concept suggesting that contaminated feed ingredients could serve as transboundary risk factors for PEDV, along with the identification of effective mitigation options.

## Background

Porcine epidemic diarrhea virus (PEDV) is an enveloped single-stranded positive sense RNA virus belonging to the Order *Nidovirales*, the family *Coronaviridae* and the genus *Alphacoronavirus* [1]. Following detection in the US swine population during May, 2013 the virus spread rapidly throughout the country [2]. During the initial outbreak, the American Association of Swine Veterinarians, the National Pork Producers Council and the USDA Center for Epidemiology and Animal Health conducted an epidemiological investigation involving case and control herds [3]. Of the 100 variables surveyed, 7 were significantly associated (*p* < 0.05) with having higher odds of acquiring PED, with all associated with the process of feeding animals [3]. In 2014, the risk of contaminated feed and feed ingredients was confirmed, when studies describing the ability of PEDV to survive for extended periods in feed (7 days in dry feed and 28 days in wet feed when stored at room temperature), and proof of concept that contaminated complete feed and feed ingredients could serve as vehicles for transmission to naïve pigs [4–6]. Shortly thereafter, the transmission of PEDV via ingestion of contaminated complete feed was validated, along with calculation of the minimum infectious dose of the virus in feed [7]. This information heightened the awareness of the need for strategies to mitigate the risk of contaminated feed, through thermal processing or chemical treatment. In regards to the latter approach, data are now available demonstrating the ability of a liquid antimicrobial product containing formaldehyde and propionic acid or a medium chain fatty acid blend to successfully degrade PEDV RNA in contaminated feed and prevent infection [8, 9].

Despite these advances at the domestic level, the risk of PEDV infection at the global level still exists, as the source of the initial PEDV introduction to the US remains unidentified. During the widespread epidemic, the role of feed in transboundary spread of the virus was downplayed, based on lack of data supporting survival of PEDV in feed ingredients over time and under conditions representative of trans-oceanic transport. Recently, extended survival of PEDV in individual feed ingredients under wintertime ambient conditions has been reported [10]. Most notably, survival of PEDV was demonstrated in soybean meal for 180 days, along with evidence of virus survival in lysine hydrochloride, choline chloride, DDGS and several porcine by-products for at least 30 days [10]. One feature that was consistent across several of these ingredients was that they are imported to the US from China. As the original PEDV detected in the US is closely related to a Chinese variant [11], it raises the question whether contaminated feed ingredients imported from China could have served as a source for viral entry to the US in 2013. Table 1 is a summary of data derived from the US International Trade Commission Harmonized Tariff Schedule website (www.hs.usitc.gov) which is a publicly available database that provides a transaction of specific trade commodities between the US and its international trading partners. These data provide insight into the types of and quantities of ingredients that entered the US from China during the height of the PED epidemic in 2013 and 2014 (G. Patterson, personal communication, May 2015). This collective information justifies renewed investigation into the transboundary risk of PEDV and the potential role that contaminated feed ingredients could play in the spread of disease during the process of trans-oceanic shipping. Therefore, the purpose of this study was to develop a model to evaluate the transboundary risk of PEDV-contaminated swine feed ingredients during a simulated shipment from China to the US, as well as test the effect of two mitigation strategies. The study was based on the hypothesis that while select non-treated ingredients could provide a protective effect on PEDV survival, mitigation would reduce risk.

**Table 1:** Summary of quantities (kg) of representative feed ingredients imported to the US from China in 2013 and 2014^a^

Ingredient	2013 (kg)	2014 (kg)
Soybeans (conventional and organic)	89,469,255	70,940,336
Lysine hydrochloride	21,469,477	5,382,454
Soybean meal (conventional and organic)	6,632,960	7,214,863
Feed-grade tetracycline	1,411,251	1,418,012
D-L methionine	377,495	1,790,280
Vitamin E	12,640	14,194
Choline chloride	11,284	23,572
Vitamin A	1156	1094
Vitamin D	1043	1052

^a^: Source = US Government Harmonized Tariff Schedule

## Methods

### Design of shipping model

#### Route and timetable

Based on the predominance of facilities processing agricultural feed ingredients for export in the eastern region of China, i.e., Shandong, Jilin, Henan, Hebei and Liaoning provinces (www.alibaba.com), and that the PEDV strain initially detected in the US most closely resembled a variant from the province of Anhui [11], the city of Beijing was designated as the starting point for the model. Here it was assumed that PEDV contamination of select ingredients would occur, either at the manufacturing plant or post-processing [4]. In an effort to determine if PEDV could be delivered in a viable state from Beijing to a major pork production region in the US, Des Moines, IA was selected as the final destination. Based on these assumptions, a commercial website (SeaRates.com) was used to develop a representative route and timetable. Specifically, it was modeled that contaminated ingredients would travel for 1 day from Beijing to the Anquing terminal in Shanghai, where they would be held for 7 days in preparation for shipment to the US. Cargo would then travel across the Pacific Ocean over a 17 day period and enter the US at the port of San Francisco. Following a 7 day period to clear customs, it would then be transported for 2 days via Interstate 80 to Des Moines, where it would remain for 3 days, for a total transit period of 37 days (Table 2).

**Table 2:** Description of locations and events involved in the 37-day shipping model period

Day	Location	Events
1	Beijing	Ingredient manufacturing & contamination
2–8	Anquing terminal, Shanghai	Transport to Shanghai, awaiting shipment to US
9–25	Pacific ocean	Departure from China, crossing the ocean
26–32	San Francisco terminal, CA, US	Entry to the US, clearing customs
33–34	Interstate 80	Transport from California to Iowa
35–37	Des Moines, IA	Storage

### Compilation of environmental data

Once the 37-day timetable had been established, it was decided to model the shipping event over the period of December 23, 2012 to January 28, 2013. This decision was based on the timing of the initial detection of PEDV in the US (April 2013) and the availability of data summarizing temperature and % RH in shipping containers traveling from Asia to the US during the period of December 31, 2012 to January 16, 2013 [12]. Using this information, we designed a temperature and % RH curve for the oceanic segment of the study. In addition, we accessed historical meteorological data for the land segments of the model using Weather Underground (www.wunderground.com) encompassing the periods of December 23–30, 2012 and January 17–27, 2013, which were paired in conjunction with the oceanic transport period. To simulate the effect of daily fluctuation we collected historical temperature data at 4 designated times each day (6 AM, 12 PM, 6 PM, 12 AM) and at 3 designated times each day for % RH (8 AM, 12 PM, 4 PM). All data were then entered into the computer of an environmental chamber used to house samples during the model shipping period.

### Processing of feed ingredients

A panel of 14 swine feed ingredients known to be imported to the US from China were selected for this study, including organic & conventional soybeans and soybean meal, lysine hydrochloride, D-L methionine, tryptophan, Vitamins A, D & E, choline chloride, two ingredient carriers (rice hulls or corn cobs) and feed grade tetracycline. In regards to the soy-based products, the guaranteed analysis of conventional meal indicated a 48 % crude protein, 1 % fat and 3 % fiber while the organic product had lower protein (44 %) and higher fat and fiber (7.5 and 6.5 %, respectively). Furthermore, the process of manufacturing organic soybean meal was void of chemical (hexane) use and no chemical fertilizer had been used during the soybean growing period. Ingredients were screened by PCR to insure a PEDV-negative status prior to the onset of the study. The treatments selected for the study included a liquid antimicrobial (LA) (SalCURB®, Kemin Industries, Des Moines, IA USA) or a medium chain fatty acid blend (MCFA). SalCURB® is a premix of aqueous formaldehyde solution 37 % (for maintenance of complete animal feeds or feed ingredients *Salmonella*-negative for up to 21 days) and propionic acid (as a chemical preservative for control of mold in feed or feed ingredients). While SalCURB® provides effective *Salmonella* control for up to 21 days, it is not approved for use by the U.S. Food & Drug Administration or the U.S. Department of Agriculture as a treatment for PEDV. The second treatment, MCFA, was a 2 % custom medium chain fatty acid blend of caproic, caprylic and capric acids, blended at a 1:1 ratio [9]. Control ingredients were treated with sterile saline.

### Sample management

Samples were organized into 1 of 4 identical batches, each representing a specific segment of the 37-day shipping period. Batch 1 (segment 1) was designed to represent the transport of contaminated ingredients from manufacturing plants in Beijing to the Shanghai Anquing terminal (day 1 post-contamination (DPC)). Batch 2, a compilation of segments 1 and 2, simulated manufacturing and delivery to Shanghai, as well as time in the Anquing terminal awaiting shipment (1–8 DPC). Batch 3, a compilation of segments 1, 2 and 3 represented time in China, the crossing of the Pacific and arrival to the US at the San Francisco, CA terminal (1–27 DPC). Finally, batch 4 was a compilation of segments 1–4, thereby representing the entire process, including transport to and storage in, Des Moines, IA (1–37 DPC). On designated days post-contamination, a batch of samples was removed from the environmental chamber and submitted for testing. Specifically, batch 1 was submitted on 1 DPC, batch 2 on 8 DPC, batch 3 on 27 DPC and batch 4 on 37 DPC. In other words, the same sample was not repeatedly opened and tested, but rather a new batch of samples was submitted on a designated day. This method of sample management would insure that all sample containers remained sealed from the time they were inoculated until the time they were tested at the lab, minimizing the risk of cross-contamination, as well as enhancing repeatability of results as several segments were replicated across batches. Finally, to increase statistical power, each of the 4 batches contained 2 replicates of each ingredient in the control group and 2 replicates of each ingredient within each treatment group, for a total of 90 samples per batch.

### Sample contamination procedures

A goal of the study was to limit the variability to the level of the ingredient; therefore, the same quantity of ingredient, the same container type and the same environmental settings were used and samples were contaminated equally. To initiate this process, 30 g of each ingredient were added to food storage containers (Oxo Tot Baby Blocks, Oxo International, El Paso, TX, USA) to simulate a shipping container [10]. Ingredients in the non-treated control group were treated with 0.1 mL of sterile saline. Ingredients in the LA group were treated with 0.1 mL of product, based on an inclusion rate of 3 kg/ton of complete feed. Ingredients in the MCFA group were treated with 0.6 g of product based on a 2 % inclusion rate. Individual treatments and saline placebo were added to the designated samples using separate tuberculin syringes. To promote proper mixing, the feed was stirred manually for 10 clockwise rotations and 10 counter-clockwise rotations using individual wooden applicator sticks per ingredient. Following mixing, each individual container was manually shaken vigorously (50 times in a 10 s period). All samples were then inoculated with 2 mL PEDV (passage 18, Ct = 17.15, total dose 491,520 FFU) and mixed as described. This quantity of PEDV was selected in an effort to provide a final mean Ct value in feed ingredient of approximately 25 (range = 19–30) following mixing, based on data from actual field cases of PEDV-contaminated feed, a challenge level used in published studies [5, 8, 10].

### Controls

For the purpose of negative controls, 30 g samples of PEDV-negative complete feed were inoculated with sterile saline. Duplicate negative controls were included in each of the 4 batches, across the control and treatment groups. For the purpose of positive controls, duplicate 5 mL samples of stock PEDV in MEM (minimum essential media, Gibco, ThermoFisher Scientific, Waltham, MA, USA) were included in containers within each batch of ingredients in both control and treatment groups.

### Sample storage

**Fig. 1:**
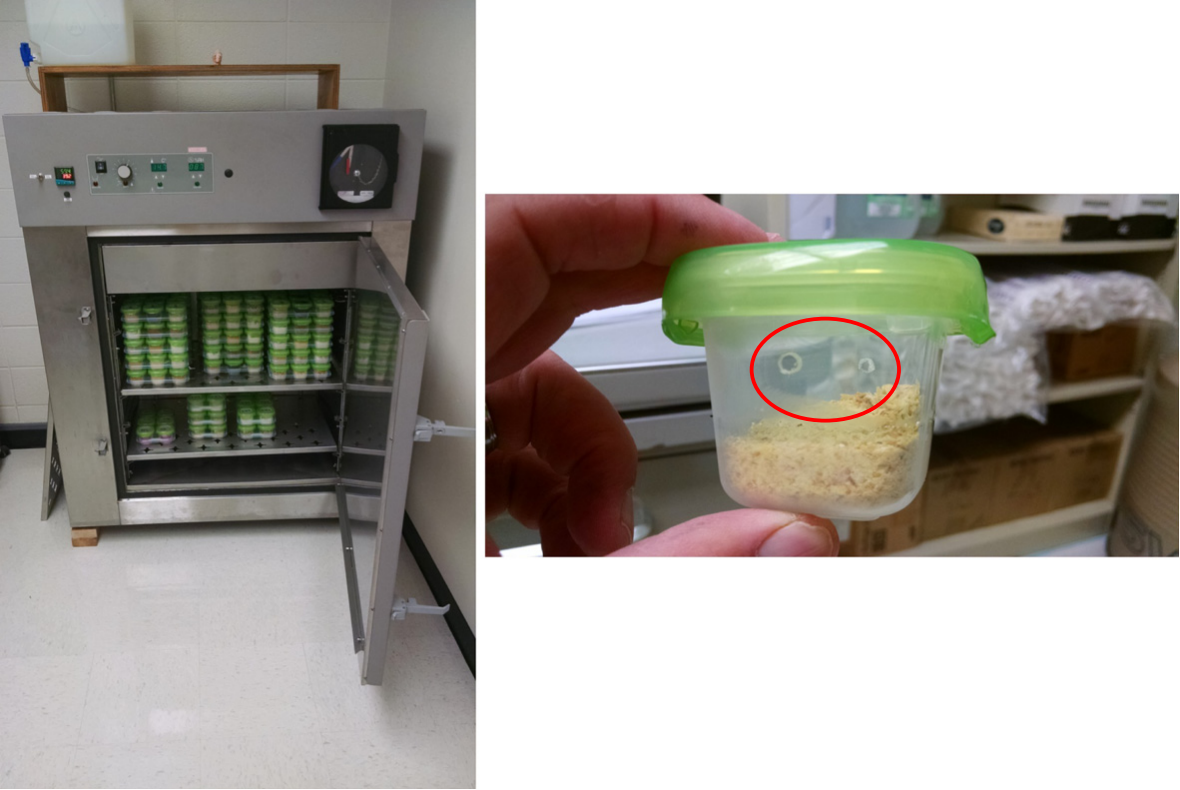
Environmental chamber and sample containers. This figure depicts the environmental chamber loaded with sample containers as well as a close-up view of a container. Note the two 0.318 cm holes (*red circle*) drilled in the container to allow for contact of the ingredient with the chamber environment

Once prepared, samples were stored in the environmental chamber. This instrument (Model 9005 L, Sheldon Manufacturing Inc., Cornelius, OR) had a programmable temperature range of 4–21^0^ C and a RH range of 40–95 %. In order for ingredients to be exposed to ambient air within the chamber, two holes, 0.318 cm in diameter were drilled into each plastic container (Fig. 1). Using the data from the historical temperature and % RH curve described above, the chamber computer was programmed to simulate fluctuation over time as previously described.

### Diagnostic procedures

All diagnostic testing was conducted using protocols developed and validated by the South Dakota State University (SDSU) Animal Disease Research and Diagnostic Laboratory (ADRDL). Samples were submitted by code to the laboratory, so personnel were blinded as to batch, treatment versus non-treatment and ingredient type. It was originally planned to test all samples by PCR and virus isolation (VI), followed by the use of swine bioassay for samples determined to be PCR positive but VI negative.

### Extraction of RNA

The MagMAX^TM^ 96 Viral Isolation Kit (Life Technologies, Waltham MA, USA) was used to obtain viral RNA from the samples, as described in the instructions provided (1836 M Revision F). A 175-μl volume of sample was used for the extraction. The magnetic bead extractions were completed on a Kingfisher96 instrument (Thermo Scientific, Waltham MA, USA).

### Real-time PCR

A commercially available real-time, single tube RT-PCR multiplex assay for the detection of PEDV, porcine deltacoronavirus (PDCoV) and transmissible gastroenteritis virus (TGEV) was used in this study per kit instruction (Tetracore, Rockville, MD, USA). Briefly, 7 μl of the extracted RNA was added to 18 μl of the master mix. The one-step real-time RT-PCR amplification conditions started with 15 min at 48 °C, followed by 2 min at 95 °C. The final cycles consisted of 5 s at 95 °C and then 40 s at 60 °C (data collection step). The program was run for 38 cycles (Cycle time) with PEDV positive results indicated at ≤ 38 cycles. Positive and negative controls were included on each run. All amplification was completed on the ABI7500 instrumentation (Austin, TX, USA).

### PEDV stock virus propagation

For PEDV propagation, Vero 76 cells (ATCC CRL-1587) were maintained in MEM plus 10 % fetal bovine serum and antibiotics. Three-day old confluent monolayers of Vero 76 cells in 150 cm^2^ flasks were washed 3 times with serum free minimum essential media (MEM) prior to inoculation. Monolayers were infected at ~0.1 moi of PEDV in MEM containing 2 *u*g/ml TPCK treated trypsin, incubated at 37 °C for approximately 48 h until obvious CPE was apparent. Flasks were frozen at −80 °C until needed.

### Virus isolation

Once feed ingredient samples were tested for PEDV via PCR, the residual samples were tested for presence of viable virus. Samples were diluted in MEM containing 2 μg/ml TPCK-treated trypsin with a starting dilution of 1:2 and were two-fold serially diluted. Diluted samples were then added to washed confluent monolayers of Vero-76 cells in 96-well plates and incubated for 1 h at 37 °C. Plates were again washed and trypsin media replaced. After 24 h at 37 °C, plates were fixed with 80 % acetone and stained with FITC conjugated mAb SD6-29 to allow visualization of infected cells. Virus concentration was determined by calculating FFU/ml based on the number of fluorescent foci present in wells at selected dilutions using a previously published method adapted to PEDV [13]. Personnel reading the plates were blinded to the type of sample and the time of sampling.

### Swine bioassay

#### Facilities and source of animals

The purpose of the swine bioassay was to determine whether viable PEDV was present in any feed ingredient sample that had tested positive on PCR but negative on VI. This study was conducted in a Biosafety Level 2+ room at the Animal Resource Wing (ARW) at South Dakota State University. All procedures involving animals throughout the study were performed under the guidance and approval of the SDSU Institutional Animal Care and Use Committee. Animals (*n* = 24, 5 day old piglets) were sourced from a PEDV-naïve herd and were tested on arrival to the ARW via blood sampling and collection of rectal swabs from each pig. Prior to animal arrival, all rooms (walls, ceilings, floors and drains) were monitored for the presence of PEDV by PCR using sampling procedures previously described [5, 8]. Piglets were housed in one of 6 stainless steel gnotobiotic units measuring 0.6 m W × 1.2 m L × 0.6 m H. Units were divided into 4 semi-isolated housing units, allowing for 4 piglets per unit with individual feeding arrangements. Flooring consisted of an open weave rubberized mat on a perforated stainless steel grate raised 10 cm for waste collection. Each unit was covered with an inflatable 20 mil plastic canopy and fitted with 2 pair of dry-box gloves for feeding and procedures inside the canopy. Each canopy was secured and sealed with duct tape and ratchet straps to the unit. Ventilation was supplied by an electric fan maintaining sufficient positive pressure inside the canopy to keep the canopy inflated above the unit. Incoming and outgoing air to each unit was HEPA-filtered. Each unit was initially sterilized using 47 % aerosolized formalin, and allowed to dissipate for 2 weeks prior to introduction of the animals. All incoming and outgoing materials needed during the study (eg. swabs, injectable medication, bleeding supplies) were passed through an air-tight stainless steel port and sterilized using 5 % peracetic acid before entering or exiting the port.

### Preparation of bioassay inoculum

The stainless steel unit served as the experimental unit; therefore, all 4 piglets in each unit received the same ingredient. To assess PEDV survivability throughout the entire model period, samples from batch 4 were tested, including both treated and non-treated ingredients. Regarding non-treated samples, all PCR-positive and VI-negative samples in batch 4 were tested. In regards to LA-treated and MCFA-treated ingredients, treated equivalents of non-treated samples that were VI or swine bioassay positive, again from batch 4 samples were tested. For preparation of the inoculums, 60 g of each specific ingredient was mixed with 50 mL of sterile PBS in a 250 mL centrifuge tube, inverted 10 times to mix and vortexed for 2 min. The suspension was then centrifuged at 5200 g for 15 min, supernatant decanted and tested by PCR prior to piglet inoculation. Each pig in the unit received 1 mL of the designated inoculum orally via syringe and observed for a 7 day period. To minimize the number of animals needed for the study, pigs that were confirmed negative after 7 DPI would be inoculated with a different ingredient. A negative control unit was included in the design, with these pigs receiving sterile saline PO.

### Piglet testing

Following inoculation, the PEDV status of each group of piglets was monitored [5, 8]. On a daily basis, ARW personnel inspected animals for clinical signs of PED and collected rectal swabs (Dacron swabs, Fisher Scientific, Franklin Lakes, NJ, USA) from each pig, starting with the negative control unit. Showers were taken upon entry to the rooms and room-specific coveralls, footwear, hairnets, gloves and P95 masks (3 M, St. Paul, MN USA) were worn. In addition, each room was ventilated individually and HEPA filtration for both incoming and outgoing air was employed per room. If clinically affected animals were observed, swabs of diarrhea and/or vomiting, in conjunction with the daily rectal swab were collected. Swabs were submitted to the SDSU ADRDL and tested by PCR. If PEDV was diagnosed in a specific unit, all animals were swabbed, humanely euthanized with intravenous sodium pentobarbital, the small intestinal tracts submitted for PCR testing, units were cleaned and sanitized as described and re-stocked with new piglets as needed.

### Data analysis

Descriptive statistics, *T*-test and ANOVA were used to analyze data, when applicable.

## Results

### Sample size

A total of 360 feed ingredient samples were used for this study.

### PCR

All feed ingredient samples were PCR negative on day 0 of the study. Successful PEDV inoculation was confirmed, as all day 1 samples were PCR-positive. Results of PCR testing of treated and non-treated ingredients on 1 and 37 DPC are summarized in Table 3. The mean Ct of LA-treated samples was 24.5 (SD = 2.4) on 1 DPC and 32.5 (SD = 3.9) on 37 DPC (*p* < 0.0001). The mean Ct of MCFA-treated samples was 24.2 (SD = 4.2) on 1 DPC and 25.5 (SD = 3.7) on 37 DPC (*p* = 0.25). The mean Ct values across non-treated ingredients on day 1 was 22.9 (SD = 2.4) and 23.1 (SD = 3.5) on day 37 (*p* = 0.34). At 1 DPC, the mean Ct values of all 3 groups (non-treated, LA-treated and MCFA-treated) were not significantly different (*p* = 0.14); however, at 37 DPC, the mean Ct of LA-treated samples was significantly higher (*p* < 0.0001) than the mean Ct of MCFA-treated and non-treated samples.

**Table 3:** Summary of PCR Ct data mean & (SD) across treated and non-treated groups on day 1 and 37 of the study

Group	Day 1 mean (SD)	Day 37 mean (SD)
Control ingredients	22.9a (2.4)	23.1a (3.5)
LA-treated ingredients	24.5a (2.4)	32.5b (3.9)
MCFA-treated ingredients	24.2a (4.2)	25.5a (3.7)

Values with different superscripts (a/b) are significantly different at *p* < 0.05

### Virus isolation

A summary of VI data is provided in Table 4. Viable PEDV was recovered from organic and conventional SBM, lysine and Vitamin D across all 4 batches of non-treated samples, including batch 3 representing entry to the US at the San Francisco terminal and batch 4, representing shipment to and storage in Des Moines. No other samples harbored viable virus beyond batch 2 (Beijing and Shanghai segments) including the PEDV stock virus control. Multiple samples were VI negative on 1 DPC. All negative control samples and all LA-treated or MCFA-treated ingredients were VI negative across all batches.

**Table 4:** Summary of mean PCR Ct and FFU/mL across all 4 batches of non-treated ingredients

Ingredient	Mean	Mean	Mean	Mean
	Ct/FFU titer	Ct/FFU titer	Ct/FFU titer	Ct/FFU titer
	Batch 1	Batch 2	Batch 3	Batch 4
Soybeans (organic)	20.76/5120	23.27/neg	26.42/neg	25.52/neg
Soybean meal (organic)	19.20/40,960	19.10/1920	20.10/800	22.55/60
Soybeans (conventional)	22.39/3840	24.18/40	28.86/neg	20.99/neg
Soybean meal (conventional)	19.75/7680	19.71/1920	19.76/5120	16.04/60
Lysine hydrochloride	18.54/1280	18.51/40	18.79/100	17.83/100
D-L methionine	22.17/2560	21.25/neg	25.03/neg	19.50/neg
Tryptophan	23.56/160	22.54/neg	25.30/neg	21.31/neg
Vitamin A	25.22/neg	22.66/neg	25.23/neg	26.85/neg
Vitamin D	22.28/3840	21.29/640	22.72/320	20.94/40
Vitamin E	28.54/neg	22.86/neg	26.15/neg	30.86/neg
Choline chloride	20.82/40	20.90/40	20.41/neg	20.77/neg
Rice hulls	24.73/neg	24.47/neg	25.93/neg	22.99/neg
Corn cobs	23.66/80	22.83/neg	24.68/neg	24.49/neg
Tetracycline	38/neg	38/neg	38/neg	38/neg
(−) control feed	38/neg	38/neg	38/neg	38/neg
Virus stock	17.15/245,760	18.86/30	21.19/neg	23.73/neg

Ingredient: Two 30 g replicates per ingredient

Mean Ct/FFU titer: Mean Ct value and FFU/mL across the 2 samples per ingredient

### Swine bioassay

Samples selected for swine bioassay testing consisted of treated and non-treated ingredients from batch 4. The non-treated ingredients tested included those which were PCR- positive and VI-negative, specifically Vitamins A & E, tryptophan, D-L methionine, soybeans (organic and conventional), and choline chloride. Viable PEDV was detected in piglets administered non-treated samples of choline chloride (Table 5). Affected animals displayed evidence of mild diarrhea, shed PEDV in feces and samples of small intestine were PCR and immunohistochemistry-positive at necropsy, with microscopic lesions of villous blunting, fusion and re-epithelialization [5]. All other samples were bioassay negative. In regards to treated ingredients, LA-treated and MCFA-treated samples of soybean meal (conventional and organic), lysine, vitamin D and choline chloride were tested. All piglets inoculated with the aforementioned LA-treated or MCFA-treated ingredients were determined to be non-infectious, as piglets remained clinically normal throughout the testing period and all rectal swab and intestinal samples were negative by PCR (Table 6).

**Table 5:** Summary of results of PCR (+)/VI (−) non-treated control feed ingredients from batch 4 tested by swine bioassay

Ingredient	Ct of inoculum	Clinical signs/rectal swabs	PCR testing of small intestine
Soybean-organic	25.52	negative	negative
Soybean-conventional	26.34	negative	negative
Vitamin A	26.86	negative	negative
Vitamin E	30.86	negative	negative
Rice hulls	22.94	negative	negative
Corn cobs	23.95	negative	negative
Tryptophan	21.31	negative	negative
D/L methionine	19.75	negative	negative
Choline chloride	20.79	positive	positive
(−) control	>38	negative	negative

**Table 6:** Summary of results of PCR (+)/VI (−) LA-treated or MCFA-treated control feed ingredients from batch 4 tested by swine bioassay

Ingredient^a^	Treatment	Ct of inoculum	Clinical signs & rectal swabs	PCR testing of small intestine
Soybean meal-conventional	LA	31.44	negative	negative
Soybean meal-organic	LA	22.38	negative	negative
Vitamin D	LA	38	negative	negative
Lysine	LA	17.83	negative	negative
Choline chloride	LA	33.70	negative	negative
Soybean meal-conventional	MCFA	23.78	negative	negative
Soybean meal-organic	MCFA	17.75	negative	negative
Vitamin D	MCFA	20.60	negative	negative
Lysine	MCFA	20.33	negative	negative
Choline chloride	MCFA	21.24	negative	negative
(−) control	Saline	38	negative	negative

^a^: Ingredients were selected based on the recovery of viable PEDV in their non-treated equivalent samples

### Environmental data

**Fig. 2:**
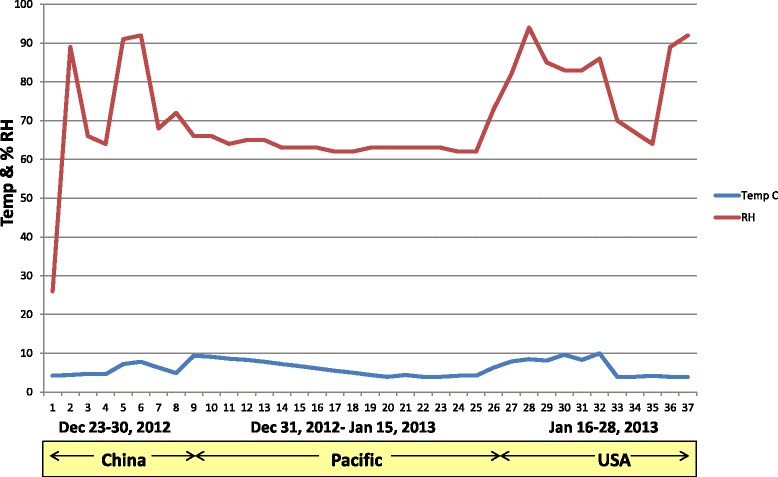
Mean daily temperature and % RH recorded during the 37-day study period. This figure depicts the summary of the environmental conditions recorded during the simulated period of December 23, 2012 to January 28, 2013

Figure 2 provides a summary of the mean daily temperature and % RH data recorded in the environmental chamber throughout the 37-day study period. For the purpose of statistical analysis, the 37-day period was divided into 4 segments: Days 1–8, representing time in China, days 9–25, representing time travelling across the Pacific, days 26–32, representing time spent in the San Francisco terminal and days 33–37, time spent during transport from California to Iowa with time in Des Moines (Table 7). In summary, mean temperature and mean % RH recorded during the San Francisco segment were significantly different (*p* = 0.0004 and *p* = 0.0025, respectively) from that recorded during the other 3 segments.

**Table 7:** Descriptive summary of temperature and % RH data per the 4 segments of the 37-day study period

Temp C0	Segment 1 (China)	Segment 2 (Pacific)	Segment 3 (SF)	Segment 4 (DSM)
	1–8 DPC	9–25 DPC	26–32 DPC	33–37 DPC
Mean	5.5	6.0	8.4	3.9
95 % CI	4.3–6.7	5.2–6.8	7.1–9.6	2.5–5.4
SD	1.4	2.0	1.2	.13
Median	4.8	5.5	8.3	3.9
Range	4.2–7.8	3.9–9.4	6.3–10	3.9–4.2
% RH	Segment 1 (China)	Segment 2 (Pacific)	Segment 3 (SF)	Segment 4 (DSM)
	1–8 DPC	9–25 DPC	26–32 DPC	33–37 DPC
Mean	71	63	84	76
95 % CI	63–79	58–69	75–92	66–87
SD	21.6	1.3	6.2	13.1
Median	70	63	83	70
Range	26–92	62–66	73–94	64–92

## Discussion

Over the course of less than 1 year (April 2013-February 2014), several novel corona viruses have been detected in the US including the PEDV prototype strain, the PEDV S INDEL strain and the porcine deltacoronavirus [14–16]. As of this writing, the route(s) of entry of these pathogens has not been identified; however, phylogenetic analyses support the possibility of a Chinese origin [11, 14–16]. Since the initial detection of PEDV in the US, feed has been proposed as a possible route of entry and widespread, rapid distribution throughout the country; however, objective data supporting this hypothesis were lacking. Therefore, the objectives of this study were to develop a model to evaluate the transboundary risk of PEDV-contaminated swine feed ingredients and test the effect of two mitigation strategies during a simulated transport event from China to the US. This approach allowed us to design a study to successfully simulate the introduction of PEDV into the US in feed ingredients; the first such objective data of its kind.

The central component of the study was the development of a model to accurately represent the conditions of a trans-oceanic shipping event to determine whether the virus could actually survive the process. Key components of the model were the selection of the proper ingredients, the calculation of an accurate timetable, and the simulation of representative environmental conditions to challenge virus viability. In regards to ingredient selection, we utilized the US Government Harmonized Tariff Schedule to include products regularly imported to the US from China, 5 of which ended up harboring viable PEDV throughout the 37-day period: vitamin D, lysine hydrochloride, choline chloride, organic and conventional soybean meal. It was interesting to note that viable virus was once again recovered from 3 ingredients, (conventional soybean meal, lysine and choline chloride). This was similar to our previous report [10], despite the fact that the 2 studies were conducted under very different environmental conditions and that laboratory personnel were blinded to sample identity/origin in both studies. This level of repeatability strengthens the conclusions drawn from both studies, thereby increasing the significance of these specific ingredients as potential transboundary risks.

Besides the consistency of the results, these ingredients were interesting for several other reasons. In regards to soybean meal, it was surprising to learn of the large quantity of soy products imported to the US from China, as the US is a major producer/exporter of soybeans and soybean meal. Using Google to search for sources of both organic and conventional soy products from China, we identified multiple soybean and soybean meal manufacturers in the eastern region of the country supplying product for agricultural use in bags, totes, containers or bulk quantities, including products designated as “organic”, targeted specifically for organic livestock feeding. Therefore, these data, in combination with our previous work demonstrating extended PEDV survival in soybean meal in cold climates [10] suggest that soy-based ingredients could be considered a significant transboundary risk for pathogen transmission, including the organic variety. Another ingredient which harbored viable virus throughout the 37-day transport period was lysine hydrochloride, a similar finding to that reported in the 30-day wintertime study [10]. Lysine is an interesting ingredient as the volume imported to the US from China is large (particularly in 2013) which could enhance the significance of this ingredient as a potential risk factor for transboundary spread of PEDV. Finally, this study brought forth new information on the risk of PEDV survival in vitamins, specifically vitamin D. During the early stages of the PED epidemic in 2013, vitamins and trace minerals were proposed as a route of viral entry, based on the volume of product imported from China. While viable PEDV was not recovered from previously tested vitamin/trace mineral mixes [10], it was speculated that this may have been due to the potentially caustic effect of the minerals on the virus. Therefore, we decided to investigate vitamins individually, and while vitamin A and E did not support virus, results were different with vitamin D. Finally, as in the previous study [10], choline chloride again proved capable of harboring viable PEDV over time, thereby raising its significance as a possible transboundary risk factor.

Along with ingredient selection, the second key component of the model was the calculation of the shipping timetable in order to truly represent this journey. Using Searates.com, we developed an accurate estimate of time required to move from the various cities and ports within the model. It did not, however, include time in port for preparation for shipping or clearance of customs. Therefore, a 7-day period of time was added at the point of each port to better represent reality (T. Schmitt, APC Inc., personal communication, 2015), increasing the calculated timetable from 23 to 37 days. This modification further challenged the viability of the virus, thereby strengthening the model.

Finally, perhaps the most novel component of the model was the development of the environmental curve which included actual temperature and % RH data from ocean containers travelling from Asia to the US [12]. This interesting project was carried out by the technical staff of the Xerox Company and involved the use of data loggers placed inside the actual containers, collecting real time temperature and % RH data as they traveled around the world. As one of the datasets involved a shipping event from Asia to the US in the months of December and January, we used this information as the centerpiece of our model and were able to reproduce the consistent trans-Pacific temperatures and % RH described in their publication along with the greater variability observed on land. Combining this with historical data from the various cities and ports using Weather Underground, we were able to develop a representative temperature and % RH curve, allowing us to program the environmental chamber accordingly (Fig. 2). One interesting observation was the rapid reduction in stock virus titer observed from the initial titer used to inoculate batch 1 (mean titer = 245,760 FFU) to that detected 8 days later in batch 2 (mean titer = 30 FFU) with the eventual loss of viability (batch 3) in the stock virus control, despite storage in MEM, an ideal medium for virus storage. This observation indicates that the environmental conditions used in the study were rigorous, thereby challenging virus survival. This was most likely due to the warmer temperatures and high % RH present in the model, conditions quite unlike the winter time project where long-term PEDV stock virus survival was observed [10]. We insured that ingredients would be exposed to the effect of the % RH by adding the holes in the sides of each container model. It also suggests the need for a supportive matrix to maintain viability in the container during shipment and that under the correct conditions, PEDVis highly susceptible to environmental pressures if left unprotected in its respective shipping container. Finally, the fact that viable PEDV was recovered from only 5 of 14 ingredients suggests that individual ingredient chemistry may provide a protective effect, potentially shielding virus from the environment and allowing it to survive and this may be particularly true of the meal form of soy versus the intact bean. In contrast, the high alkalinity of feed grade tetracycline (pH 13) apparently degraded the RNA immediately post-contamination, resulting in PCR-negative readings in all 4 batches.

In an effort to assess the potential for chemical mitigation of ingredient risk, two interventions, the liquid antimicrobial SalCURB® and the medium chain fatty acid blend were included in the design. While we had validated the LA in previous studies [8, 10], this was our first opportunity to assess the MCFA option. While the effect of the LA on PEDV RNA degradation over the course of the study was significantly different than the response to MCFA, both products appeared to have equivalent effect on virus viability. This outcome supports the validity of chemical mitigation as a means to reduce the risk of PEDV in feed ingredients stored under conditions modeling the trans-oceanic voyage, as well as provides options for treatment.

In addition to an improved understanding of risks and mitigation, other strengths of the study included an experimental design where the only variables in the study were the individual ingredients, and the presence or absence of the treatment. Specifically, we inoculated equal amounts of each ingredient with an equivalent quantity of virus in an attempt to mimic published viral loads associated with field cases of PEDV in feed. All ingredients were stored in identical models of shipping containers, exposed to the same environment and tested in a single laboratory, involving consistent, trained personnel and validated assays for the detection of PEDV. We organized samples in a manner to prevent cross-contamination by insuring that the individual sample storage containers were never opened from the time of PEDV inoculation until testing occurred at the laboratory. While this may have resulted in some variability of the PCR assay since a new batch was submitted each time, the fact that our negative control samples remained free of contamination throughout the entire project validated these protocols. Finally, we used multiple metrics (PCR, VI and bioassay) to document viral load across a total of 360 samples, exceeding our previous work.

However, as with all studies there were both strengths and limitations. For example, we were limited to 14 ingredients primarily due to cost; however, the decision to use similar ingredients across the 2 studies enhanced replication and confidence in the data. While we were limited in the number of replicates, each batch contained 2 replicates/ingredient/batch; resulting in 8 replicates per ingredient. Furthermore, these results were derived from gram quantities of feed and may not directly equate to the vast quantity of tonnage used in actual swine production or feed manufacturing facilities. The bioassay protocol, while helpful at identifying ingredients containing low levels of virus was costly and difficult to carry out. As we evaluated only one shipping route over a defined period of time under a specific set of environmental conditions, this does not allow for extrapolation of results across other climates, other routes or over longer periods of time. Finally, as this was an experiment and did not take place in China, we could not provide proof on how the contamination of ingredients could actually take place. However, from our experiences in the US, we could speculate that contamination of feed could take place through the inclusion of contaminated ingredients or through contact with the virus in the environment, either at the farm or at the mill [6, 10]. As PEDV dispersion throughout the mill setting appears to be widespread, contact with ingredients within a milling storage facility seems logical [17].

In closing, under the conditions of this study, we provided the first proof of concept data indicating that a subset of contaminated feed ingredients could successfully transport live PEDV into the US using a novel transboundary shipping model. These are the first objective data suggesting that feed ingredients could potentially serve as vehicles for pathogen transfer between countries, as well as providing a plausible explanation regarding how PEDV was introduced to the US in 2013. This study also provides insight on how the onset of organic farming could serve to elevate risk of pathogen introduction into the US, secondary to the entry of organic soy products. This study also introduces a model which could enhance further transboundary research efforts, possibly employing surrogate viruses (bovine viral diarrhea virus for swine fever virus or Seneca Valley virus for foot-and-mouth disease virus) to test feed-related risks for other foreign animal diseases, further the investigation of the risk of organic ingredients, along with the continuing validation of mitigation strategies. With this information, it is hoped that veterinarians, feed industry experts and government officials will work together to develop a comprehensive plan of ingredient management to reduce global risk. It is time for discussions on how to adequately treat feed, how to increase biosecurity at the port level, as well as where to purchase biosecure ingredients, with the end result being a lowered incidence of feed-related disease transmission around the world.

## Conclusions

These results indicate the ability of PEDV to survive in specific feed ingredients under modeled conditions simulating shipment from China to the US. This is the first proof of concept suggesting that contaminated feed ingredients could serve as transboundary risk factors for PEDV, along with the identification of effective mitigation options.

### Availability of supporting data

The data set(s) supporting the results of this article is included within the article.

## Abbreviations

PEDV: porcine epidemic diarrhea virus
PED: porcine epidemic diarrhea
Ct: Cycle threshold
PCR: polymerase chain reaction
ARW: Animal Resource Wing
MEM: minimal essential media
SBM: soybean meal
LA: liquid antimicrobial
